# Supplementary material for: Glycerol suppresses glucose consumption in trypanosomes through metabolic contest
Source: PLoS Biol. 2021 Aug 13;19(8):e3001359. doi: 10.1371/journal.pbio.3001359 (PMC8386887; doi:10.1371/journal.pbio.3001359)

**S1 Fig.** Growth curves of the parental (WT) and the tetracyclic-induced *^RNAi^*GK.i cell lines maintained in presence of 10 mM glucose and glycerol (+Glc, +Glyc), 10 mM glucose (+Glc, -Glyc), 10 mM glycerol (-Glc, +Glyc), or none of them (-Glc, -Glyc). Cells were maintained in the exponential growth phase (between 10^6^ and 10^7^ cells/ml), and cumulative cell numbers reflect normalization for dilution during cultivation.


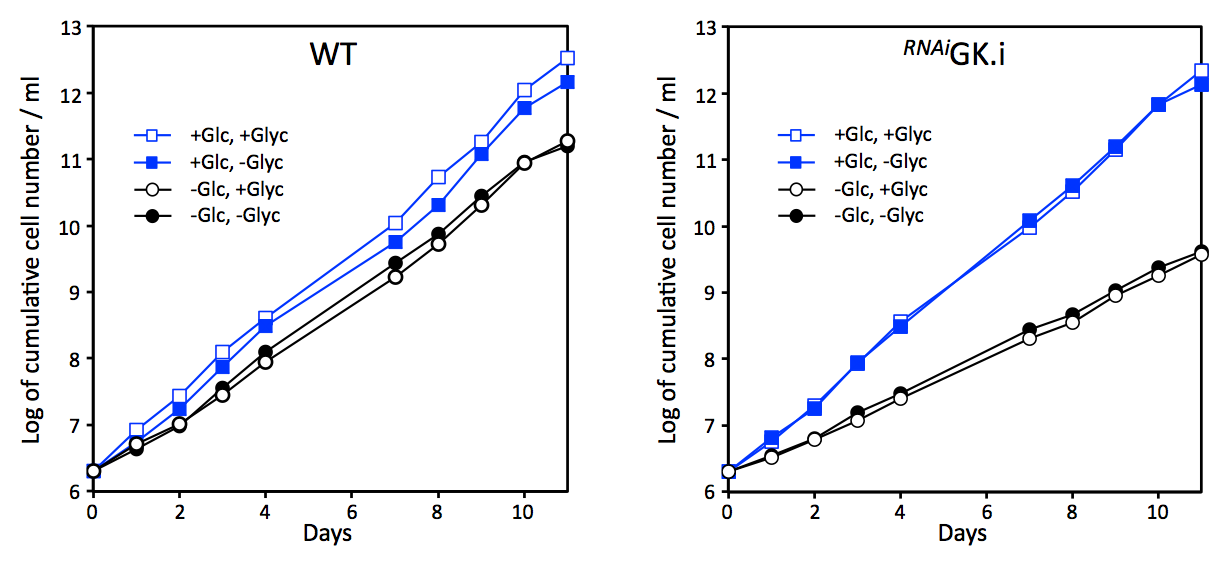

Supplement: S1 Fig — Cells were maintained in the exponential growth phase (between 106 and 107 cells/mL) and cumulative cell numbers reflect normalization for dilution during cultivation. (DOCX) [file pbio.3001359.s001.docx]
