# Supplementary material for: Glycerol suppresses glucose consumption in trypanosomes through metabolic contest
Source: PLoS Biol. 2021 Aug 13;19(8):e3001359. doi: 10.1371/journal.pbio.3001359 (PMC8386887; doi:10.1371/journal.pbio.3001359)

**S2 Fig.** HK activity in different combinations (indicated in the table below the graph) of total cell extracts from the parental (WT) and the *^RNAi^*GK.i cell lines. The amounts of HK remain the same in all samples, while the amounts of GK present in the parental samples are diluted with the GK-depleted *^RNAi^*GK.i samples. The HK and GK activities were determined in the presence of both glucose and glycerol, as performed in the Glc/Glyc conditions (see Fig 2B).


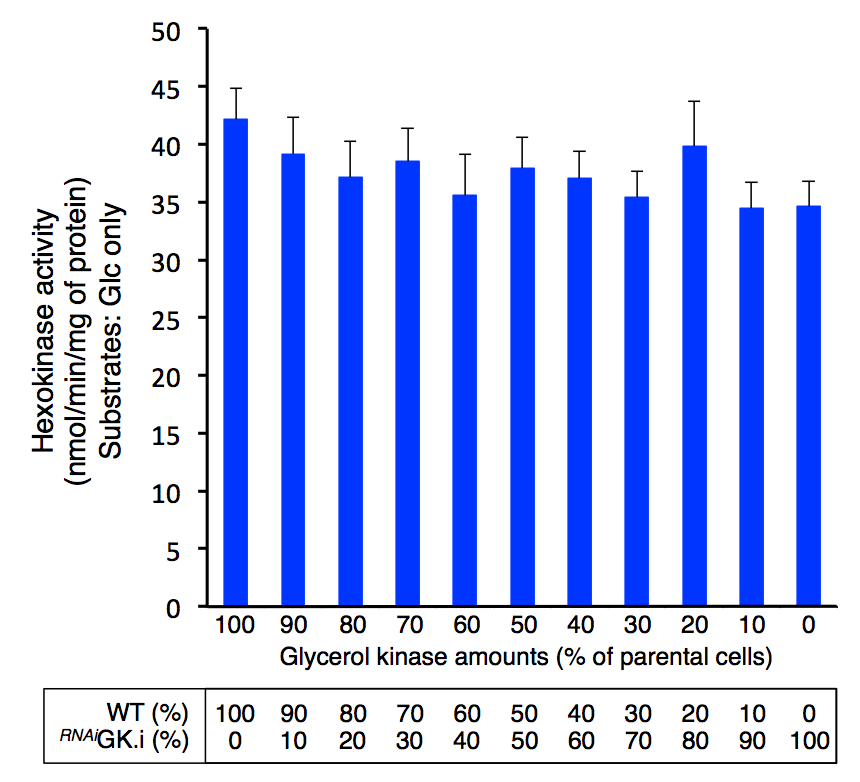

Supplement: S2 Fig — The amount of HK remains the same in all samples, while the amount of GK present in the parental sample is diluted with the GK-depleted RNAiGK.i sample. The HK and GK activity were determined in the presence of both glucose and glycerol, as performed in the Glc/Glyc conditions (see Fig 2B). (DOCX) [file pbio.3001359.s002.docx]
