# Supplementary material for: Glycerol suppresses glucose consumption in trypanosomes through metabolic contest
Source: PLoS Biol. 2021 Aug 13;19(8):e3001359. doi: 10.1371/journal.pbio.3001359 (PMC8386887; doi:10.1371/journal.pbio.3001359)
Supplement: S1 Table — The extracellular PBS medium of trypanosomes incubated in the presence of 4 mM of 1 or 2 carbon sources was analyzed by 1H-NMR spectroscopy to detect and quantify excreted end products. Data supporting the results described in this table can be found at https://zenodo.org/record/5075637#.YORd2B069yA. (DOCX) [file pbio.3001359.s003.docx]

**S1 Table.** Excreted end-products from metabolism of [U-^13^C]-glycerol and/or glucose by the parental (EATRO1125.T7T), *^RNAi^*GK.ni and *^RNAi^*GK.i procyclic *T. brucei* cell lines. The extracellular PBS medium of trypanosome incubated in the presence of 4 mM of one or two carbon sources was analyzed by ^1^H-NMR spectroscopy to detect and quantify excreted end-products. Data supporting the results described in S1 Table can be found at <https://zenodo.org/record/5075637#.YORd2B069yA>.

|  |  | | | | | | | | | | | | |
| --- | --- | --- | --- | --- | --- | --- | --- | --- | --- | --- | --- | --- | --- |
| Cell line | Carbon source(s) metabolized^a^ |  |  | nmol/h/mg of protein | | | | | | | | | |
|  |  |  | N^b^ |  |  |  |  |  |  |  |  |  |  |
|  |  |  |  | Acetate | | Succinate | | Lactate | | Alanine | | TOTAL | |
|  |  |  |  |  |  |  |  |  |  |  |  |  |  |
| Parental^c^ | **[U-^13^C]-Glycerol** |  | 9 | **1386** | **± 192.9** | **1123** | **± 205.3** | **ND**^d^ | | **ND** | | **2509** | **± 231.6** |
|  |  |  |  |  |  |  |  |  |  |  |  |  |  |
| Parental^c^ | **[U-^13^C]-Glycerol** |  | 6 | **1445** | **± 209.4** | **811** | **± 101.3** | **ND** | | **ND** | | **2256** | **± 17.8** |
|  | Glucose |  |  | 44 | ± 15.4 | 27 | ± 15.0 | ND | | ND | | 71 | ± 10.7 |
|  |  |  |  |  |  |  |  |  |  |  |  |  |  |
| Parental^c^ | Glucose |  | 6 | 1727 | ± 115.3 | 401 | ± 89.2 | 9 | ± 16.7 | 19 | ± 36.0 | 2156 | ± 153.7 |
|  |  |  |  |  |  |  |  |  |  |  |  |  |  |
|  |  |  |  |  |  |  |  |  |  |  |  |  |  |
| *^RNAi^*GK.ni | **[U-^13^C]-Glycerol** |  | 6 | **591** | **± 149.3** | **215** | **± 67.5** | **ND** | | **ND** | | **806** | **± 88.2** |
|  |  |  |  |  |  |  |  |  |  |  |  |  |  |
| *^RNAi^*GK.ni | **[U-^13^C]-Glycerol** |  | 6 | **429** | **± 97.6** | **155** | **± 44.2** | **ND** | | **ND** | | **584** | **± 133.5** |
|  | Glucose |  |  | 1239 | ± 69.7 | 718 | ± 216.6 | 60 | ± 10.4 | 10 | ± 11.3 | 1968 | ± 300.2 |
|  |  |  |  |  |  |  |  |  |  |  |  |  |  |
| *^RNAi^*GK.ni | Glucose |  | 6 | 1558 | ± 242.6 | 633 | ± 61.7 | 61 | ± 8.4 | 17 | ± 8.6 | 2208 | ± 257.1 |
|  |  |  |  |  |  |  |  |  |  |  |  |  |  |
|  |  |  |  |  |  |  |  |  |  |  |  |  |  |
| *^RNAi^*GK.i | **[U-^13^C]-Glycerol** |  | 6 | **35** | **± 14.4** | **8** | **± 6.2** |  | **ND** |  | **ND** | **43** | **± 20.5** |
|  |  |  |  |  |  |  |  |  |  |  |  |  |  |
| *^RNAi^*GK.i | **[U-^13^C]-Glycerol** |  | 6 | **59** | **± 5.4** | **8** | **± 0.6** |  | **ND** |  | **ND** | **66** | **± 5.6** |
|  | Glucose |  |  | 1537 | ± 214.2 | 678 | ± 106.3 | 78 | ± 8.5 | 10 | ± 11.5 | 2226 | ± 260.3 |
|  |  |  |  |  |  |  |  |  |  |  |  |  |  |
| *^RNAi^*GK.i | Glucose |  | 6 | 1599 | ± 349.1 | 732 | ± 109.0 | 75 | ± 8.7 | 23 | ± 3.1 | 2353 | ± 451.8 |
|  |  |  |  |  |  |  |  |  |  |  |  |  |  |
|  |  |  |  |  |  |  |  |  |  |  |  |  |  |

*^a^* Incubation conditions (carbon sources added to the PBS medium). *^b^* Number of duplicates. *^c^* the measured consumption of glucose was 1200 ±98 and 58 ±55 nmol/h/mg of protein in the presence of glucose alone and glucose + [U-^13^C]-Glycerol, respectively. *^d^* Non detectable
