# Supplementary material for: Glycerol suppresses glucose consumption in trypanosomes through metabolic contest
Source: PLoS Biol. 2021 Aug 13;19(8):e3001359. doi: 10.1371/journal.pbio.3001359 (PMC8386887; doi:10.1371/journal.pbio.3001359)
Supplement: S2 Table — The extracellular PBS medium of trypanosomes incubated in the presence of 4 mM of 1 or 2 carbon sources was analyzed by 1H-NMR spectroscopy to detect and quantify excreted end products. Data supporting the results described in this table can be found at https://zenodo.org/record/5075637#.YORd2B069yA. (DOCX) [file pbio.3001359.s004.docx]

**S2 Table.** Excreted end-products from metabolism of [U-^13^C]-glycerol and/or glucose by the parental (EATRO1125.T7T), *^RNAi^*GKcst, *^RNAi^*GKcst/*^OE^*GKrec.ni and *^RNAi^*GKcst/*^OE^*GKrec.i cell lines, grown in the presence of glucose or glycerol (Glyc). The extracellular PBS medium of trypanosome incubated in the presence of 4 mM of one or two carbon sources was analyzed by ^1^H-NMR spectroscopy to detect and quantify excreted end-products. Data supporting the results described in S2 Table can be found at <https://zenodo.org/record/5075637#.YORd2B069yA>.

|  |  | | | | | | | | | | | | |
| --- | --- | --- | --- | --- | --- | --- | --- | --- | --- | --- | --- | --- | --- |
| Cell line^a^ | Carbon source(s) metabolized^b^ |  |  | nmol/h/mg of protein | | | | | | | | | |
|  |  |  | n^c^ |  |  |  |  |  |  |  |  |  |  |
|  |  |  |  | Acetate | | Succinate | | Lactate | | Alanine | | TOTAL | |
|  |  |  |  |  |  |  |  |  |  |  |  |  |  |
| Parental | **[U-^13^C]-Glycerol** |  | 9 | **1386** | **± 192.9** | **1123** | **± 205.3** | **ND**^d^ | | **ND** | | **2509** | **± 231.6** |
|  |  |  |  |  |  |  |  |  |  |  |  |  |  |
| Parental | **[U-^13^C]-Glycerol** |  | 6 | **1445** | **± 209.4** | **811** | **± 101.3** | **ND** | | **ND** | | **2256** | **± 17.8** |
|  | Glucose |  |  | 44 | ± 15.4 | 27 | ± 15.0 | ND | | ND | | 71 | ± 10.7 |
|  |  |  |  |  |  |  |  |  |  |  |  |  |  |
| Parental | Glucose |  | 6 | 1727 | ± 115.3 | 401 | ± 89.2 | 9 | ± 16.7 | 19 | ± 36.0 | 2156 | ± 153.7 |
|  |  |  |  |  |  |  |  |  |  |  |  |  |  |
|  |  |  |  |  |  |  |  |  |  |  |  |  |  |
| Parental (Glyc) | **[U-^13^C]-Glycerol** |  | 3 | **1357** | **± 194.2** | **1110** | **± 97.5** | **ND** | | **ND** | | **2467** | **± 100.4** |
|  |  |  |  |  |  |  |  |  |  |  |  |  |  |
| Parental (Glyc) | **[U-^13^C]-Glycerol** |  | 3 | **1336** | **± 128.5** | **890** | **± 169.7** | **ND** | | **ND** | | **2226** | **± 51.0** |
|  | Glucose |  |  | 89 | ± 40.3 | 49 | ± 16.2 | ND | | ND | | 138 | ± 34.7 |
|  |  |  |  |  |  |  |  |  |  |  |  |  |  |
| Parental (Glyc) | Glucose |  | 3 | 1413 | ± 138.1 | 690 | ±104.7 | 63 | ± 10.5 | 100 | ± 56.1 | 2266 | ±192.4 |
|  |  |  |  |  |  |  |  |  |  |  |  |  |  |
|  |  |  |  |  |  |  |  |  |  |  |  |  |  |
| *^RNAi^*GKcst | **[U-^13^C]-Glycerol** |  | 3 | **33** | **± 8.0** |  | **ND** | **ND** | | **ND** | | **33** | **± 8.0** |
|  |  |  |  |  |  |  |  |  |  |  |  |  |  |
| *^RNAi^*GKcst | **[U-^13^C]-Glycerol** |  | 3 | **60** | **± 6.4** | **12** | **± 1.5** | **ND** | | **ND** | | **72** | **± 6.5** |
|  | Glucose |  |  | 1596 | ± 92 | 558 | ± 38.1 | 22 | ± 1.3 | 41 | ± 19.9 | 2217 | ± 145.4 |
|  |  |  |  |  |  |  |  |  |  |  |  |  |  |
| *^RNAi^*GKcst | Glucose |  | 3 | 1602 | ± 126.7 | 629 | ± 33.5 | 27 | ± 2.7 | 27 | ± 3.3 | 2285 | ± 159.7 |
|  |  |  |  |  |  |  |  |  |  |  |  |  |  |
|  |  |  |  |  |  |  |  |  |  |  |  |  |  |
| *^RNAi^*GKcst/*^OE^*GKrec.ni | **[U-^13^C]-Glycerol** |  | 3 | **1113** | **± 67.7** | **1330** | **± 84.0** | **ND** | | **ND** | | **2443** | **± 151.3** |
|  |  |  |  |  |  |  |  |  |  |  |  |  |  |
| *^RNAi^*GKcst/*^OE^*GKrec.ni | **[U-^13^C]-Glycerol** |  | 3 | **1092** | **± 73.6** | **934** | **± 46.2** | **ND** | | **ND** | | **2026** | **± 119.4** |
|  | Glucose |  |  | 134 | ± 14.5 | 64 | ± 15.5 |  | ND |  | ND | 208 | ± 25.6 |
|  |  |  |  |  |  |  |  |  |  |  |  |  |  |
| *^RNAi^*GKcst/*^OE^*GKrec.ni | Glucose |  | 3 | 1730 | ± 162.5 | 722 | ± 79.6 | 34 | ± 3.6 | 29 | ± 3.3 | 2515 | ± 247.1 |
|  |  |  |  |  |  |  |  |  |  |  |  |  |  |
|  |  |  |  |  |  |  |  |  |  |  |  |  |  |
| *^RNAi^*GKcst/*^OE^*GKrec.i | **[U-^13^C]-Glycerol** |  | 3 | **1312** | **± 44.9** | **1425** | **± 56.9** | **ND** | | **ND** | | **2738** | **± 101.6** |
|  |  |  |  |  |  |  |  |  |  |  |  |  |  |
| *^RNAi^*GKcst/*^OE^*GKrec.i | **[U-^13^C]-Glycerol** |  | 3 | **1299** | **± 130.4** | **1077** | **± 67.3** | **ND** | | **ND** | | **2376** | **± 197.2** |
|  | Glucose |  |  | 23 | ± 15.9 | 28 | ± 13.4 | 15 | ± 1.4 |  | ND | 66 | ± 30.3 |
|  |  |  |  |  |  |  |  |  |  |  |  |  |  |
| *^RNAi^*GKcst/*^OE^*GKrec.i | Glucose |  | 3 | 1767 | ± 81.8 | 647 | ± 8.0 | 29 | ± 1.0 | 32 | ± 1.6 | 2475 | ± 85.4 |
|  |  |  |  |  |  |  |  |  |  |  |  |  |  |

*^a^* The parental cells (EATRO1125.T7T) were cultivated several days in SDM79 containing 10 mM glucose or in glucose-depleted SDM79 containing 10 mM glycerol and 50 mM GlcNAc (Glyc), before incubation in PBS, and the mutant cell lines were cultivated in the absence of glycerol. *^b^* Incubation conditions (carbon sources added to the PBS medium). *^c^* Number of duplicates. *^d^* Non detectable.
