# Supplementary material for: Glycerol suppresses glucose consumption in trypanosomes through metabolic contest
Source: PLoS Biol. 2021 Aug 13;19(8):e3001359. doi: 10.1371/journal.pbio.3001359 (PMC8386887; doi:10.1371/journal.pbio.3001359)
Supplement: Attachment — Submitted filename: Answer_Reviewers.pdf [file pbio.3001359.s005.pdf]

Reviewer #1:
[identifies herself as Lena Pernas]
How a eukaryotic cell chooses a carbon substrate is a timely and important question that is relevant in almost every aspect of eukaryotic biology from cancer cell metabolism (how does a cancer cell choose its metabolite) to infection metabolism (which carbon sources do pathogens choose). In this manuscript, the authors present an exciting new framework for considering how a eukaryotic cell might choose a carbon substrate. Using trypanosomes, the authors describe a 'metabolic contest' between glycerol kinase and hexokinase, which phosphorylate glycerol and glucose respectively. They claim that this 'metabolic contest' depends on i) the sequestration of metabolic pathways in the same subcellular compartment; ii) competition for the same substrate, ATP; and iii) unbalanced activity between the competing enzymes. While the presented framework for choosing metabolic substrates is novel (and very logical), the authors have only convincingly demonstrated the relevance of iii) unbalanced activity between glycerol kinase and hexokinase. It remains unclear whether i) the sequestration of glycerol kinase and hexokinase in the glycosome is required for substrate competition, as well as whether levels of ATP influence (ii) the outcome of the metabolic contest between glycerol kinase. It would be important to convincingly establish these points if they are part of the 'metabolic contest' framework put forth. To address this, perhaps the authors can:

1) Regarding i) the sequestration of metabolic pathways in the same subcellular compartment: can the authors express a cytosolic version of glycerol kinase to determine whether this also limits hexokinase activity? According to the framework the expected result would be that glucose and glycerol would be simultaneously metabolized. Add different levels of ATP and assess whether carbon utilization is in anyway affected

Answer: To address this question, we re-expressed a glycosomal or cytosolic GK in the constitutive ^{RNAi}GK background (^{RNAi}GKcst). To do so, the recoded *GK* gene, resistant to the RNAi construct, containing or not the last 9 residues coding for the PTS1 glycosomal targeting motif (GKrec and GKrec-akl, respectively), was introduced in the ^{RNAi}GKcst cell line, which constitutively down regulated expression of the endogenous *GK* genes (the constitutive absence of GK in the ^{RNAi}GKcst cell line is shown in panel A of the following figure). The data are presented in the following figure.

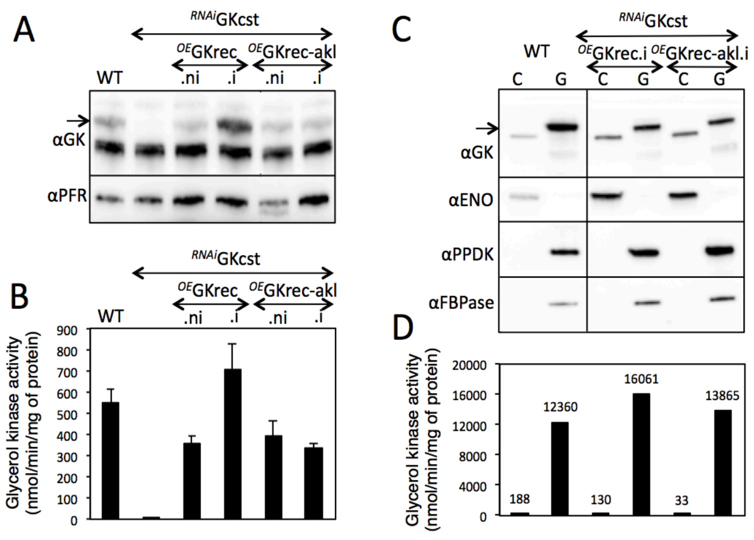

GK re-expression in the ^{RNAi}GKcst background. *(A) Expression of the recoded GK containing (GKrec) or not (GKrec-akl) the glycosomal targeting sequence (PTS1) in the ^{RNAi}GKcst background, in the presence (.i) or the absence (.ni) of tetracycline, was controlled by Western blotting on total cell extracts using immune sera against GK (αGK) and paraflagellar rod (αPFR) as control. The parental ^{RNAi}GKcst (constitutive RNAi) cell line and the WT cells were also analysed. (B) GK activity normalized with malic enzyme activity was determined for the same cell lines. (C) The subcellular localization of the recoded GK was determined by Western blotting on glycosomal (G) and cytosolic (C) fractions using immune sera against GK, the cytosolic enolase and the glycosomal PPDK and FBPase. (D) the glycerol kinase activity was determined in the cytosolic and glycosomal fractions of the same cell lines.*

It was expected that the resulting tetracycline-induced ^{RNAi}GKcst/^{OE}GKrec.i and ^{RNAi}GKcst/^{OE}GKrec-akl.i cell lines would express GKrec exclusively in the glycosomes or in the cytosol, respectively. Importantly, since a high level of GK expression is required for "metabolic contest", we used a strong expression vector in order to reach at least the expression level of the native GK. This goal was achieved for the control ^{RNAi}GKcst/^{OE}GKrec.i cell line, as shown by western blot (panel A) and GK activity (panel B), with GKrec expressed exclusively in the glycosomes (Panel C) and, as expected,

restoration of "metabolic contest" with the absence of glycolysis in the presence of glycerol (Fig 3C of the corrected manuscript).

In contrast, after three unsuccessful attempts while each transfection routinely generate at least a dozen of clones, we only got a single ^{RNAi}GKcst/^{OE}GKrec-akl.i cell line, which turned out to constitutively express an active GK (panels A and B) in the glycosomes (panels C and D). A PCR analysis of the ^{RNAi}GKcst/^{OE}GKrec-akl cell line suggested that the pLew100-GKrec-akl plasmid recombined with an endogenous *GK* copy (the genome contains 5 tandemly arranged *GK* genes per loci) to generate a GKrec copy containing the C-terminal "AKL" PTS1 glycosomal targeting motif, which is consistent with the glycosomal localization of GKrec and its constitutive expression. We concluded that expression of high amounts of GK in the cytosol is lethal for procyclic trypanosomes, as previously observed by down-regulating expression of PEX14, a protein involved in glycosomal protein import (Haanstra et al., PNAS, 2008:17718–17723). Therefore, this biological lock does not allow us to answer the question raised by the reviewer.

However, we took advantage of the production and analysis of the ^{RNAi}GKcst/^{OE}GKrec.ni cell line, that still showed the glycerol-to-glucose preference although its GK activity was reduced as compared to wild-type cells, to include a new paragraph emphasizing that glycerol down-regulates GK expression but does not affect glycerol-to-glucose preference.

2) Regarding ii) ATP competition: can the authors supplement cultures with higher levels of ATP and determine whether this promotes dual usage by glycerol kinase and hexokinase? Or visualize ATP in the glycosome using previously established ATP sensors?

Answer: Extracellular ATP cannot enter into trypanosomes, however, we developed a new assay to measure intraglycosomal changes in ATP levels depending on growing conditions to address the reviewer's comment. Indeed, we used fluorescence resonance energy transfer (FRET)-based indicators for ATP that were composed of the ε subunit of the bacterial F_oF_1-ATP synthase sandwiched by the cyan- and yellow-fluorescent proteins (CFP and YFP, respectively) and named ATeam (Imamura et al. in 2009 [ref 24]). In the ATP-bound form, the ε subunit retracts to bring the two fluorescent proteins close to each other, which increases FRET efficiency and allows detection of changes in ATP level. To focus on the glycosomal ATP level, the ATeam cassette was fused to the N-terminal extremity of a Myc-tagged glycosomal protein, *i.e.* glycerol-3-phosphate dehydrogenase (GPDH; EC 1.1.1.8; Tb927.8.3530), which contains a C-terminal peroxisomal targeting motif (PTS1). We previously used this approach to target a cytosolic protein exclusively to the organelle [ref 25]. Upon tetracycline-induction, the anti-GPDH and anti-Myc immunsera recognized a 111 kDa protein corresponding the expected size of the ATeam-Myc-GPDH recombinant protein (Fig 4C). Immunofluorescence analyses showed that the ATeam-Myc-GPDH recombinant protein was located in glycosomes, as confirmed by the colocalization of the ATeam-Myc-GPDH signal detected (YFP) and that of the aldolase (Aldo) glycosomal marker (Fig 4D, top panel), as well as the colocalization of CFP and YFP FRET signals forming glycosomal-like images (Fig 4D, bottom panel). FRET efficiency was significantly decreased in the ^{OE}ATeam-Myc-GPDH.i cell incubated in the presence of glycerol compared to glucose conditions, which indicated that glycerol metabolism induced a reduction of the intraglycosomal ATP level compared to the standard glucose conditions (Fig 4E). This was confirmed by an increased of the CFP fluorescence lifetime corresponding to a FRET decrease (Fig 4F). This reduced glycerol-induced glycosomal ATP level is in line with our "metabolic contest" model, since the glycerol-to-glucose preference is optimal with ATP levels lower than 0.6 mM, which corresponds to the measured glycosomal ATP concentration. These new data have been included in a dedicated paragraph entitled "GK and HK compete for glycosomal ATP" and in Figure 3 (Fig 4B-F in the corrected version of the manuscript).

In addition:

1) Given that glycerol enters via diffusion / facilitated diffusion, the authors might also want to discuss whether cellular permeability of a substrate might also be a key aspect of such a metabolic aspect

Answer: Indeed, Jeacock et al. (PLoS Pathog, 2017) demonstrated that trypanosomes uptake glycerol through aquaglyceroporins, which are encoded by three different *AQP* genes. To address this point, we have included the following sentence at the end of the paragraph describing abolition of "metabolic

contest" by knocking down GK expression: "It is worth mentioning that knocking down the 10 identical *GK* genes is far easier than doing the alternative experiment consisting on knocking down/out the three different aquaglyceroporin genes (*AQP1-3*) responsible for glycerol uptake in *T. brucei* [18]"

Reviewer #2:
The present article examines carbon source choice in T. brucei. I found it quite interesting that this parasite prefers glycerol to glucose (at least at certain developmental stages) and that even in the presence of glucose, glycerol is used for g6P synthesis and that glycerol effectively suppresses glucose utilization. Reading the paper, however, I couldn't quite tell if all of these observations were novel. Indeed, when I searched the same author's work, I saw a 2018 PLoS pathogens paper with sentence in abstract " In addition, metabolomic analyses by mass spectrometry showed that, in the absence of glucose, 13C-labelled glycerol was incorporated into hexose phosphates through gluconeogenesis," which I thought was 1 of the most interesting findings in reading this paper.
Answer: this reviewer refers to the manuscript "Glycerol supports growth of the Trypanosoma brucei bloodstream forms in the absence of glucose: Analysis of metabolic adaptations on glycerol-rich conditions", in which we indeed described gluconeogenesis from glycerol, in the **absence** of glucose. This observation was expected, since in the absence of glucose, G6P, which is the precursor of essential pathways, has to be produced from alternative carbon sources, here glycerol. However, in this submitted manuscript, we demonstrated that gluconeogenesis from glycerol occurs even in the **presence** of glucose, which has not been reported so far in any microorganism.

Thus, it seems like some of the core substance of Fig 1 is not novel.
Answer: again, gluconeogenesis from glycerol, in the **presence** of glucose, has never been reported so far.

This is a problem because I found the other figures less compelling. The key claim in the Title is "Metabolic Contest" which is mainly shown by competing activities in lysate assay in Fig 2. But this lysate assay involves ATP consumption (presumably down to more or less zero). No dynamics of product production or ATP consumption are shown. Of course, 1 fast and abundant enzyme that consumes ATP will use up all the ATP in a lysate assay, so that another slower or less abundant enzyme that uses ATP never gets a chance. But for this mechanism to operate in cells, where ATP is regenerated, it requires the fast enzyme to persistently deplete ATP in the compartment. This would also be deleterious for the activity of the fast enzyme, and sets up a different kind of competition that depends substantially on Km (really Vmax/Km, as opposed to mainly just Vmax in case where ATP starts out abundant and gets depleted to zero) and proximity to whatever the limited ATP source is.
Fig 3 and 4 did little to illuminate or support the main theme to me, so while some interesting regulation (or "non-regulation" simply by mass action, as the authors hint at) is going on, I don't have much confidence what the actual mechanism is for glycerol suppression of glucose metabolism in cells. I would encourage the authors to carry out dynamic measurements for their lysate assays, prove whether suppression of hexokinase flux occurs only when ATP has been depleted …
Answer: To address these two related comments, we determined in our *in vitro* enzymatic assay the ATP concentrations required to prevent HK activity in the presence of excess GK, in order to understand the molecular basis of the "metabolic contest". HK activities of trypanosome extracts were followed over time of incubation in the presence of glycerol and different amounts of ATP. In the presence of GK activity, HK activity is maintained until ATP concentrations reach the mM range (1.1-1.5 mM depending on the initial amounts of ATP, *i.e.* 1.2 to 3 mM). However, in the presence of lower amounts of ATP, such as 0.6 mM, no HK activity was detected, while GK was active during 1.5 min until all ATP is consumed. These data demonstrate that HK activity is inhibited by GK excess in the presence of physiological amounts of ATP (0.6 mM in glycosomes, ref [19]), however, at higher ATP concentrations, HK is active until ATP levels drop below 1.5 mM. It is noteworthy that *T. brucei* HK and GK have the same affinity for ATP (*K*m = 0.28 mM and 0.24 mM, respectively, ref [21,22]), suggesting that the GK-to-HK preference at ATP concentrations below 0.6 mM is primarily due to the large excess of GK activity. We have included these data in the result section (paragraph entitled "GK and HK compete for glycosomal ATP") and in Figure 3 (Fig 4A in the corrected version of the

manuscript).

… and use some mechanism to probe ATP in the relevant compartment in the presence of glycerol, glucose, or both (maybe a genetically engineered sensor would be good for this).
Answer: We have successfully addressed this question by expressing the ATeam ATP sensor fused with a glycosomal protein for targeting to glycosomes, as described above to answer the reviewer 1's comment.

They might also consider some quantitative modeling of glycerol and glucose use vs. compartmental ATP in a situation where ATP is regenerated.
Answer: The Bakker's group previously developed a kinetic model of trypanosome glycolysis to understand the role of compartmentalization of this pathway in the glycosomes and to determine the contribution of glucose transport to control the glycolytic flux (Bakker et al., 1997, JBC; Bakker et al., 1999, JBC; Bakker et al., 1999, PNAS; Haansdra et al., 2098, PNAS). In the course of these analyses they addressed the effect of glycerol on glycolysis in anaerobic conditions, since a high amount of glycerol is produced to maintain the glycolytic flux in the absence of oxygen. The experimental data were confirmed by the kinetic model, which was also used to study *in silico* the effect of glycerol on glycolysis in aerobic conditions. These data have been briefly mentioned in Bakker's manuscripts. In Bakker et al. (PNAS, 1999), they mentioned in the discussion section: "by using the model of trypanosome glycolysis (Bakker et al., 1997, JBC; Bakker et al., 1999, JBC), it was demonstrated that this inhibition (*inhibition of glucose uptake by glycerol*) may be fully explained by metabolic effects, notably the competition of glucose and glycerol for glycosomal ATP". To address the point raised by the reviewer, we have included in the new paragraph the following sentence, "competition between HK and GK for glycosomal ATP was previously anticipated by using a kinetic model of trypanosome glycolysis [23]".
