# Supplementary material for: Glycerol suppresses glucose consumption in trypanosomes through metabolic contest
Source: PLoS Biol. 2021 Aug 13;19(8):e3001359. doi: 10.1371/journal.pbio.3001359 (PMC8386887; doi:10.1371/journal.pbio.3001359)
Supplement: Attachment — Submitted filename: Answer_Reviewers.pdf [file pbio.3001359.s007.pdf]

Based on the review, we will probably accept this manuscript for publication, provided you satisfactorily address the remaining points raised by the reviewers. Please also make sure to address the following data and other policy-related requests.
Answer: We thank the reviewer and the editor for accepting our manuscript for publication in PLoS Biology.

a) We agree with the reviewer's suggestion about the title, and strongly suggest that you change it to the following slightly modified version: "Glycerol suppresses glucose consumption in Trypanosomes through metabolic contest."
Answer: We agree that the proposed title is more appropriate and have been included accordingly.

b) Please provide a blurb, according to the instructions in the submission form.
Answer: The following sentence (blurb) has been included in the first page of the manuscript: Trypanosomes developed "metabolic contest" for the regulation of nutrient utilization based on the competition between two enzymes for a common substrate, while most microorganisms use the well known "catabolite repression".

c) Please address my Data Policy requests below; specifically, please supply numerical values underlying Figs 1BCDFGH, 2BCDE, 3ABCD, 4AEFGHI, 5ABC, and cite the location of the data clearly in each relevant Fig legend.
Answer: We replaced "Fig 1F-H" by "Figs 1FGH" (page 6) and "Fig 4E-F" by "Figs 4EF" (page 12). Data supporting the results described in all figures can be found at https://zenodo.org/record/5075637#.YORd2B069yA with doi:10.5281/zenodo.5075637. This sentence has been included in each figure legend.
